# A common type system for clinical natural language processing

**DOI:** 10.1186/2041-1480-4-1

**Published:** 2013-01-03

**Authors:** Stephen T Wu, Vinod C Kaggal, Dmitriy Dligach, James J Masanz, Pei Chen, Lee Becker, Wendy W Chapman, Guergana K Savova, Hongfang Liu, Christopher G Chute

**Affiliations:** 1 Mayo Clinic, Rochester, Rochester, MN, USA; 2 Childrens Hospital Boston and Harvard Medical School, Boston, MA, USA; 3 University of Colorado at Boulder, Boulder, CO, USA; 4 University of California, San Diego, San Diego, CA, USA

**Keywords:** Natural Language Processing, Standards and interoperability, Clinical information extraction, Clinical Element Models, Common type system

## Abstract

**Background:**

One challenge in reusing clinical data stored in electronic medical records is that these data are heterogenous. Clinical Natural Language Processing (NLP) plays an important role in transforming information in clinical text to a standard representation that is comparable and interoperable. Information may be processed and shared when a *type system* specifies the allowable data structures. Therefore, we aim to define a common type system for clinical NLP that enables interoperability between structured and unstructured data generated in different clinical settings.

**Results:**

We describe a common type system for clinical NLP that has an end target of deep semantics based on Clinical Element Models (CEMs), thus interoperating with structured data and accommodating diverse NLP approaches. The type system has been implemented in UIMA (Unstructured Information Management Architecture) and is fully functional in a popular open-source clinical NLP system, cTAKES (clinical Text Analysis and Knowledge Extraction System) versions 2.0 and later.

**Conclusions:**

We have created a type system that targets deep semantics, thereby allowing for NLP systems to encapsulate knowledge from text and share it alongside heterogenous clinical data sources. Rather than surface semantics that are typically the end product of NLP algorithms, CEM-based semantics explicitly build in deep clinical semantics as the point of interoperability with more structured data types.

## Background

Electronic medical records (EMRs) hold immense promise for improving both practice and research. Area 4 of the Strategic Healthcare IT Advanced Research Project (SHARP 4, or SHARPn) aims to reuse data from the EMR, analyzing records on a large scale – an effort known as high throughput phenoytyping. Many large-scale applications are dependent on high throughput phenotyping, such as characterizing the prevalence of a disease, or finding patients who fit the criteria for a clinical or epidemiological study. A prerequisite is that information across patients, areas of practice, and institutions must be comparable and interoperable. SHARP 4 has adopted Intermountain Healthcare’s Clinical Element Models (CEMs) as the standardized format for information aggregation and comparison. This representation is both concrete and specific, yet allows for some of the ambiguity that is inherent in clinicians’ explanation of a clinical situation.

However, a significant amount of information in the EMR is not available in any form that could be easily mapped to CEMs. It is no surprise that health care professionals prefer to record a significant proportion of their information in the format of human language, rather than more structured formats like CEMs. Therefore, Natural Language Processing (NLP) techniques are necessary to tap into this extensive source of clinical information. The goals for NLP in SHARPn are to normalize information from clinical text into the structured CEMs, which are more conducive to computation at a large scale.

A *type system* specifies data structures that may be used for the processing and sharing of information. In this work, we define a type system whose key innovation is that it implements a comprehensive model of clinical semantics types, based on CEMs. This deep semantic target is integrated with a comprehensive brush of types for existing language analysis tools, allowing the type system to be used for arbitrary clinical use cases and to be compatible with a diversity of underlying NLP approaches. Therefore, we call it a *common* type system, with highly structured output semantics intended to interoperate with structured data from the EMR. Additionally, NLP components that use the type system will be interchangeable with each other. The type system was initially designed for practical NLP use in UIMA (Unstructured Information Management Architecture [1]), which allows for flexible passing of input and output data types between components of an NLP system.

Our preliminary work [2] has been fully adopted by Mayo Clinic’s popular open source NLP tool, cTAKES (clinical Text Analysis and Knowledge Extraction System [3]), as of cTAKES 2.0. The current work presents a full picture of the type system, alongside a thorough example of how the type system may be used in practice to house SHARPn-style CEMs. Our description is consistent with the implementation in cTAKES v2.5 (http://sourceforge.net/projects/ohnlp/files/cTAKES/).

### UIMA and type systems

UIMA was originally designed by IBM to process text, speech, or video [1]. Here, we concern ourselves with clinical text as our domain of input. Each clinical document that is processed within UIMA is automatically marked up (annotated) by components called Analysis Engines, which are often arranged in a pipeline. Analysis Engines may be interchanged if they solve the problems and annotate the data in the same way.

However, the structure of the markup must be defined in order for Analysis Engines to be interoperable. A *type system* defines the structure for possible markup, providing the necessary data types for downstream components to make use of partially processed text, and gives upstream components a target representation for markup data. For example, after sentence detection, a document will have identified types called Sentence; after tokenization, a document will have identified types called WordToken. Each type may have associated *features*, which give additional information about the structure. For example, a WordToken could have an associated part-of-speech VB (verb). In this article, we will use “feature” and a related term, “attribute,” interchangeably.

The data are then passed between Analysis Engines in an efficient framework, the Common Analysis Structure (CAS), which includes the original document, the results of the analysis, and indices for efficient searching of these results. To facilitate outputs from and inputs to UIMA, the CAS can also be efficiently serialized and de-serialized. With this architecture, UIMA enables interoperability between Analysis Engines and encourages a development of “best-of-breed” components.

All UIMA-based techniques will have a type system [4-6], and other tools (such as the General Architecture for Text Engineering (GATE) [7]) typically have analogous schemata for artifacts. Most of these type systems encode the same basic information as our common type system, including types for storing text span annotations, syntax, and document annotations. In a few cases, types and features (e.g., a List structure) were introduced into our common type system based on an analysis of these systems.

The reported work within SHARP 4 is an attempt to provide a common type system for diverse NLP use cases centering around clinical texts and domain semantics. Therefore, the our most significant contributions are the extensive semantic model based on CEMs and the separation between textual semantic types and referential (referring to the real-world) semantic types. These contributions enable a development of diverse technologies that serve different clinical use cases.

### Deep semantics with clinical element models

From a linguistic perspective, this common type system embeds a deep semantic representation analogous to those that have been used in the computational semantics and dialogue systems communities [8,9]. It distinguishes between semantic content that refers to real-world phenomena and the textual surface form used to communicate the semantics. However, we might expect the impact of a mature, deep semantic representation for Clinical NLP to be much greater, since this is an enabling technology for many downstream tasks like patient classification and high-throughput phenotyping. Designing the type system to account for these deep semantics as output gives room for technological innovations around the CEM structure.

In addition to providing a well-developed semantic data model, the common type system provides a wide range of data types to bridge from text and linguistic structure to deep semantics. In doing so, it allows for downstream access to both the more raw, textual data types and the deeper semantic representation.

For SHARPn, six “core CEMs” have been identified and are under continuing development: Anatomical Sites, Diseases and Disorders, Signs and Symptoms, Procedures, Medications, and Labs. A specific CEM for “cough” would have the same basic structure as any other Signs and Symptoms, as defined by the Signs and Symptoms core CEM:

<<cetype kind="statement" name="CoughAssert" xmlns="">

<key code="Assertion_KEY_ECID" /><data domain="CoughType_VALUESET_ECID" type="cwe" />

…

<qual card="0-M" name="periodicity" type="Periodicity" />

<qual card="0-1" name="course" type="Course" />

<qual card="0-1" name="severity" type="Severity" />

…

<mod card="0-1" name="subject" type="Subject" />

<mod card="0-1" name="negationInd" type="NegationInd" />

<mod card="0-1" name="uncertainty" type="Uncertainty" />

<att card="0-1" name="observed" type="Observed" />

<att card="0-1" name="reportedReceived" type="ReportedReceived" />

<att card="0-1" name="verified" type="Verified" />

</cetype>

The basic structure of a CEM consists of a type, a key, and a value choice; qualifiers, modifiers, and attributions give further detail. The Type is a coded value that represents the constraints to which all instances of a given model will conform (e.g., *cwe* – coded with extensions, or *pq* – physical quantity). The key is a coded value for the real world concept that is important to what an instance is attempting to describe (e.g., since we are modeling text, *Assertion* is a common key). Finally, the value choice is a choice between a “data property” or “items,” where the former is a derivative of the HL7 version 3 data type “ANY,” and the latter is a sequence of one or more clinical elements (e.g., “CoughType” is a data property, constraining data values).

A qualifier captures information that does not change the meaning of the value choice (e.g., the “periodicity” of a cough). A modifier adds information that changes the meaning of the value choice (e.g., “negationInd” may reverse the asserted CoughType). An attribution defines an action and the contextual information for the action (e.g., “observed” gives the context of the Cough).

In the end, this work reports, alongside other NLP-relevant types, a casting of CEMs from the above structure into a UIMA type system.

## Methods

To define our common type system, we began with the types in the cTAKES v1.1 type system. These types had primarily been developed ad hoc while doing information extraction tasks in UIMA. They were therefore loosely arranged around a typical information extraction pipeline (including components such as sentence detection, tokenization, lemmatization, named entity recognition (NER), and negation/status detection). We analyzed a number of existing UIMA type systems to find useful types that caused us to augment or modify the original type definitions. We eventually modified existing types and categorized them into 4 groupings: Utilities, Text Spans, Syntax, and Text Semantics. We also created 3 new groupings: Structured Data, Relations, and Referential Semantics.

In this breakdown of 7 groupings, we have ensured that the resulting semantic model distinguishes clearly between text semantics and referential semantics. This distinction is important for several reasons. The NLP task of Named Entity Recognition may define semantics in terms of a semantic type or even a mapping to an ontology code (possible with text semantics types), but additional structure is necessary when populating post-coordination or attribute templates (possible with referential semantics types). Furthermore, there is no clear interpretation for the results of coreference resolution with only text semantics. Especially in the clinical context, a chain of related text mentions does not define an event; for example, if a patient has a severe cough with sputum, but coreferring mentions in the text do not mention the sputum, this does not mean that there is no sputum. Finally, deep semantic structure is necessary because it enables interoperability with all other types of medical information. With this CEM-based referential semantic model, structured data and NLP results can then be used together seamlessly in high-throughput phenotyping efforts.

The effort in the type system definition was that of creating types for the referential semantics grouping, in which our goal was to represent the core CEM templates. Notably, we created a separate type for each of the 6 core CEMs. The distinction between qualifiers, modifiers, and attributions in the core CEMs was dropped, and all were considered to be features. Common features between the core CEMs were moved into the Element supertype. The text semantic model was adjusted to complement the referential semantic model. Outside of the semantic model, new types were also created for standard NLP tasks, such as constituent and dependency parsing, relation extraction, and temporal relations.

Overall, the type system design attempts to follow best practices for UIMA type systems, as recommended by UIMA’s original developers. These have to do with ease and completeness of representation, as well as computational cost. For example, defining a subtype is an efficient way to subset data because indices for types are reliably calculated. However, we do not put locally used (component-specific) types in the CAS, as there is no garbage collection in UIMA and extra types only bloat the type system. Where possible, we assumed existing standards for NLP tasks (e.g., types for constituent parses should be consistent with Treebank II).

## Results

We present the SHARPn common type system in its entirety, as released with cTAKES 2.5. This type system is an extensive update of the cTAKES type system, with modifications, restructuring, and additions. While there are carry-over types from previous iterations of the cTAKES type systems, use case specific types were dropped, as they would be local to custom components at the end of a pipeline.

The type system’s seven groups correspond to 7 namespaces. We will thoroughly describe these groups, focusing especially on syntax, text semantics, relations, and referential semantics with examples to highlight our contribution.

Notationally, we introduce types with small caps and in their namespaces (e.g., textspan.Sentence), but refer to them informally by just capitalizing just the first letter (e.g., Sentence). Features (attributes) of each type are introduced with colons (e.g., Sentence:begin). Using the equivalent data structures outside of UIMA do not strictly require these namespaces or groupings. Also, in the following figures, dark gray boxes indicate types are in a different namespace, but are necessary to fully describe the inheritance of another type (e.g., uima.tcas.Annotation).

### Structured data types

**Figure 1:**
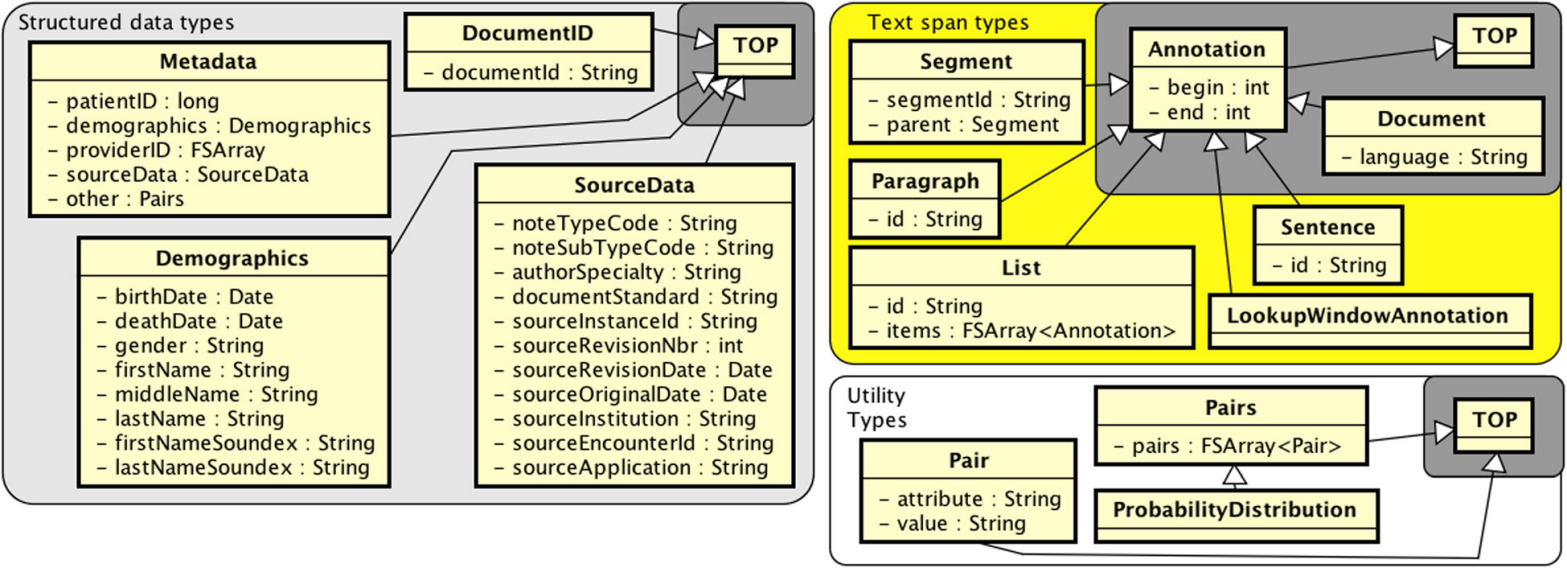
**Types and features for 3 namespaces.** Structured data types, Utility types, and Text span types. Dark gray background coloring indicates types that are not in the namespace but are included to show inheritance. Arrows indicate inheritance.

Unstructured clinical text is generated in the wider context of clinical settings. Structured data can provide useful information about the clinical context, both to improve information extraction and to more easily retrieve results. Structured data types are shown on the left side of Figure 1.

### Utility types

This minimal grouping (bottom right, Figure 1) replaces a type from cTAKES v1.1 called Property with the util.Pair type. Each Pair:attribute corresponds with some Pair:value, and a util.Pairs type stores multiple ones. This is tacitly a brute-force implementation of a probability distribution as well, hence the util.ProbabilityDistribution type. More efficient means of defining probability distributions are possible, but this is not easily done in a generalizable type system to be used with UIMA.

### Text span types

Text span types shown on the top-right side of Figure 1 are typically discourse-level subdivisions of a text document into organizational components. The type Document spans a whole document and subsumes other text span types. In UIMA implementations of the type system, this Document type is by default available from the UIMA itself (hence the gray box); non-UIMA implementations would need Document to be introduced explicitly. Other types break down the text into spans of decreasing size: textspan.Segment (e.g., sections of a clinical note), textspan.Paragraph, textspan.List, and textspan.Sentence.

### Syntactic types

**Figure 2:**
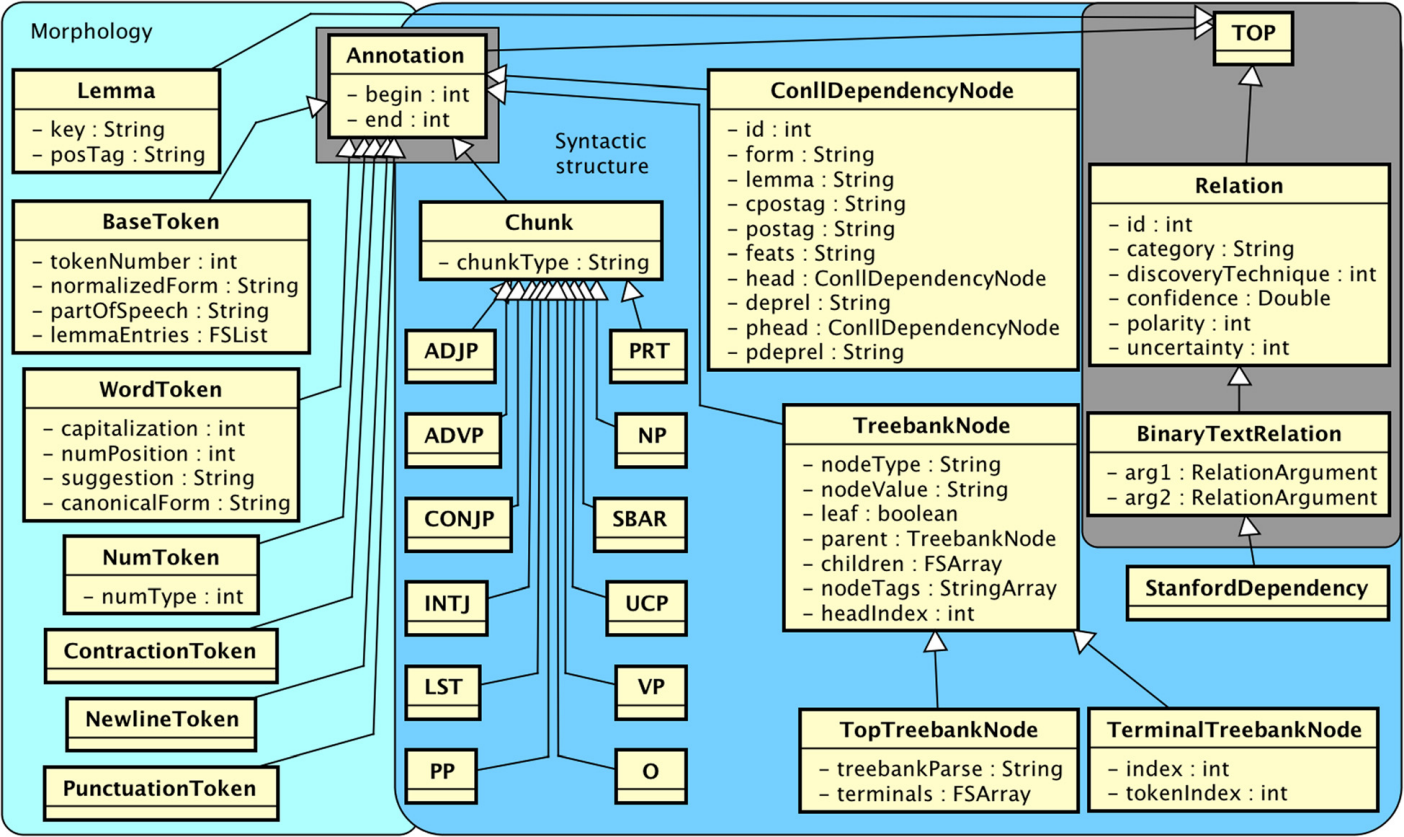
The syntax namespace: types for morphology and syntax.

The syntax namespace in Figure 2 shows two major groupings: Morphology (light blue) and Syntactic Structure (darker blue). The Morphology grouping deals with the internal characteristics of words and borders between words. It centers around Syntax.BaseToken, a supertype for word, punctuation, symbol, newline, contraction, or number tokens. It includes parts of speech in BaseToken:partOfSpeech, which are grammatical categories, e.g., noun (NN) or preposition (IN) that use Penn Treebank tags^a^ with a few additions. BaseToken:normalizedForm stores a final normalized form, including processes such as lemmatization and abbreviation expansion.

Because syntactic processing has been well studied in NLP, the common type system adopts established standards for the majority of its grammatical types. Phrase-level syntactic categories found by the process of shallow parsing (chunking) are stored in Syntax.Chunk types. The Chunk:chunkType feature draws from phrasal Penn Treebank II categories. Many of these phrasal tags (syntax.ADJP, syntax.NP, syntax.VP, etc.) are included as child types to syntax.Chunk for ease of indexing.

More robust syntactic structure is embodied in syntax.ConllDependencyNode, a dependency parse node spanning a single BaseToken. This follows the CONLL-X Shared Task [10] format with 10 features. Dependency parses are produced sentence-by-sentence. Each node of a parsed sentence refers to its syntactic head, i.e., another ConllDependencyNode pointed to from ConllDependencyNode:head.

Like shallow parses, deep (constituent) parses can be represented consistently with Penn Treebank II standards [11]. Here, we use Syntax.TreebankNode and two convenience subtypes, Syntax.TerminalTreebankNode and Syntax.TopTreebankNode. The syntactic constituent can be found in TreebankNode:nodeType or TreebankNode:nodeValue.

Stanford dependencies [12] are formally triples of two tokens plus the relationship between them. Thus, they are represented as Syntax.StanfordDependency, binary relation types that relate heads to dependents, where the inherited StanfordDependency:arg1 is the head, StanfordDependency:arg2 is the dependent, and StanfordDependency:category stores the type of relation (e.g., nsubj).

### Textual semantic types

**Figure 3:**
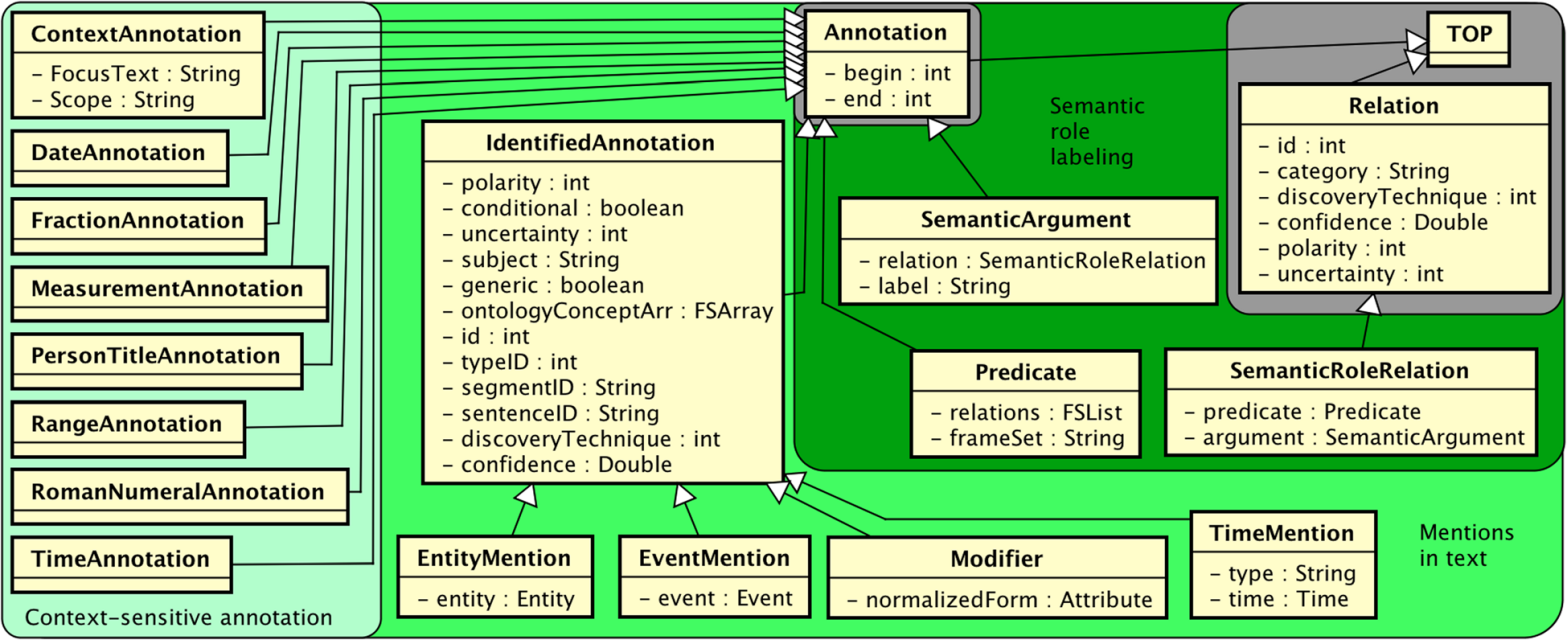
The textsem namespace: spanned types for shallow semantics.

The main intent of textual semantic types is for spans of text to house a shallow sense meaning or function. These types are shown in Figure 3, progressing from simpler meaning on the left to more complex meaning on the right.

### Context-sensitive annotations

On the left of Figure 3, several simple types indicate spans of text that are of use in clinical context. The Textsem.ContextAnnotation type is a lightweight, spanned type that tags the context surrounding an entity or event. It may be used for quick iteration and search. The feature ContextAnnotation:Scope has example values like “left,” “right,” or “both,” indicating on which side of a ContextAnnotation:FocusText the entity or event might be found.

A set of subtypes of Annotation (Date, Fraction, Measurement, PersonTitle, Range, RomanNumeral, Time) is included for quick indexing. These are frequently found in clinical texts that report laboratory and procedure values, proper names with titles, and temporal information.

### Mentions in text

The middle section of Figure 3 has types that are similar to Named Entities and Events as defined by the Automatic Content Extraction and Message Understanding Conference (MUC-7) tasks, and emerging ISO standards. Notably, Textsem.IdentifiedAnnotation is a span of text that must be discovered, generalizing traditional Named Entities. Children of IdentifiedAnnotation are intended to bridge the gap between a text-centric/shallow-semantic view of the data, versus a concept-centric/deep-semantic view of the data. For example, “Patient has pain in the abdomen… pain increases with pressure” has two (co-referring) text spans for “pain,” but would have one unified referential semantic representation. Thus, subtypes of IdentifiedAnnotation each have an attribute that refers to the deeper semantic representation, to be described in the Referential semantics section.

For a shallow semantic representation, IdentifiedAn-notation:ontologyConceptArr allows an array of hypotheses about how the text should be mapped to an ontology. The array allows (but does not require) users to utilize techniques that separate word sense disambiguation (WSD) from the initial recognition. Other features give some measure of how the annotation is presented in context – IdentifiedAnnotation:polarity (stated with negation), IdentifiedAnnotation:conditional (e.g., “… should return *if* any rash occurs”), IdentifiedAnnotation:uncertainty (stated with doubt), IdentifiedAnnotation:subject (stated in reference to an entity, e.g., the patient), and IdentifiedAnnotation:generic (stated without a real-world instance, e.g., “lupus clinic”).

Other administrative features are included as well. The semantic type (e.g., drugs, disorders, etc.) may be stored in IdentifiedAnnotation:typeID; the segmentID and sentenceID features provide an indexing to the section and sentence within which the annotation is found; the means of discovering the IdentifiedAnnotation is stored in IdentifiedAnnotation:discoveryTechnique; and for automatic extraction methods, a confidence score may be stored in IdentifiedAnnotation:confidence.

Two subtypes of primary importance are Textsem.EntityMention and Textsem.EventMention. The former is an IdentifiedAnnotation that refers to a real-world entity (embodied in EntityMention:entity). Similarly, EventMentions are spans that refer to real-world events as captured by EventMention:event. We have built in a distinction that Events have a temporal nature (see refsem.EventProperties) whereas Entities do not. In clinical text, the large majority of relevant mentions are temporally active.

Other IdentifiedAnnotations may not refer to entities or events, but may instead represent attributes of those elements. For example, if a medication is prescribed for one month, this may be discovered in text as “1 month,” “a month,” “1 mo,” etc. We store this text in textsem.Modifier and point it to a normalized version in refsem.Attribute. Time annotations have their own subtype as well, textsem.TimeMention, since the time stamp of an event is an important attribute that may or may not be set from text.

### Semantic role labeling

Semantic role labeling [13] is a standard NLP task [14] that gives a shallow representation of the semantics of a sentence. We follow the conventions of PropBank [15] in defining the semantic roles. TextSem.SemanticArgument is used for the arguments in predicate-argument structures (SemanticRoleRelations). The SemanticArgument:label features should contain the type of semantic role (e.g., ARG0, ARGM) that this argument has (with respect to the predicate). The predicate and other information about the semantic role are available through the SemanticRoleRelation type.

We follow PropBank standards for TextSem.Predicate with a few clinical additions. Predicates are typically verbs and may participate in semantic role relations (accessible via Predicate:relations). The corresponding TextSem.SemanticRoleRelation type embodies the semantic role labeling output as a binary Relation between SemanticRoleRelation:predicate and SemanticRoleRe-lation:argument.

### Referential semantic types

**Figure 4:**
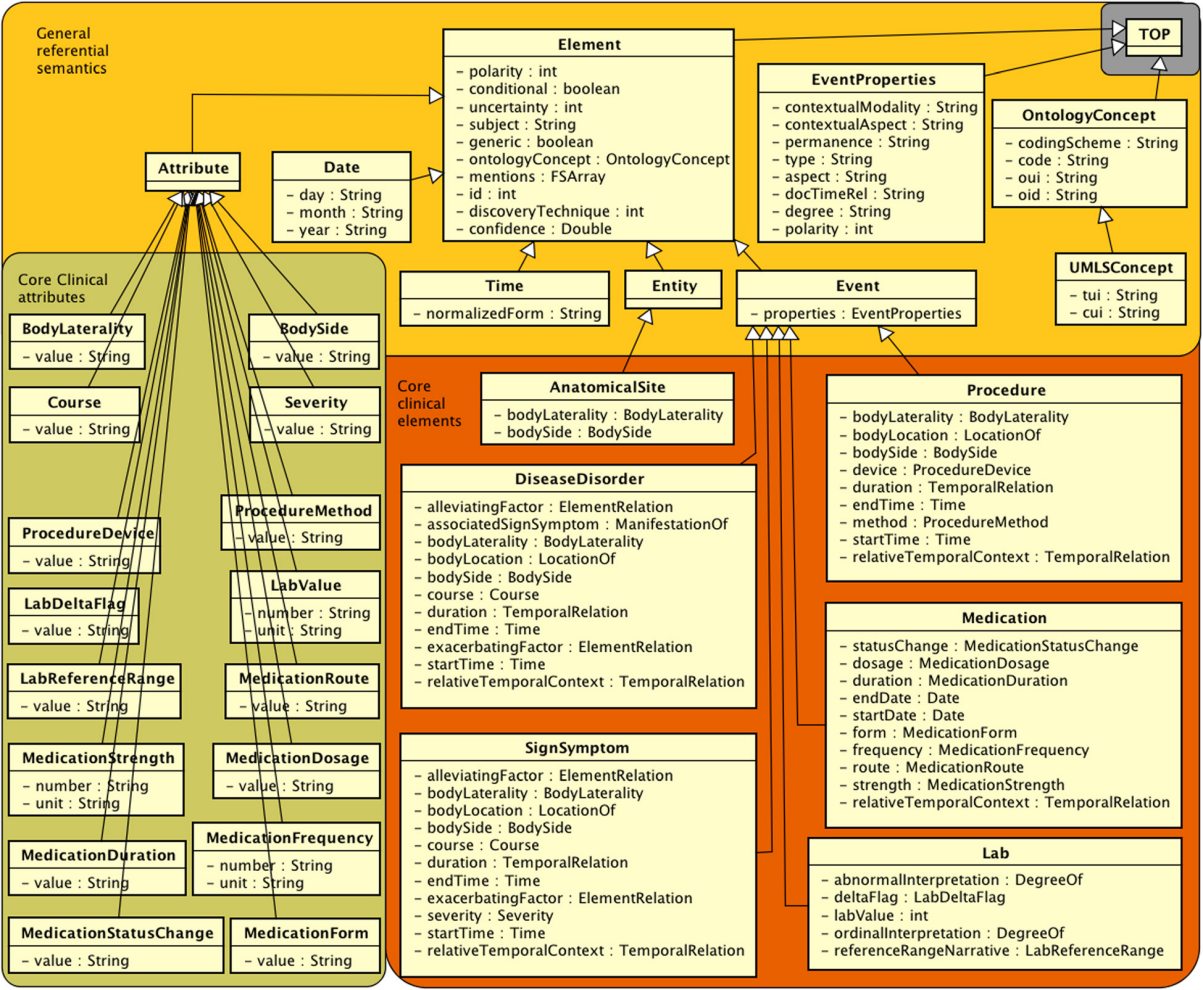
The refsem namespace, with deep semantic types and a model of core CEMs.

The types in Figure 4 are a deep semantic representation that are meant to refer to something in the real world. Unlike the text semantics grouping, referential semantic types do not inherit from Annotation, and they are thus document-level objects (assuming that one document is processed per CAS).

### General referential semantics

We begin referential semantics with the refsem.OntologyConcept and refsem.UMLSConcept types. Ontologies (e.g., the Systematized Nomenclature of Medicine – Clinical Terms, or SNOMED-CT) provide precise, expert-curated semantic specifications for concepts. IdentifiedAnnotations and Elements may point to these normalized concept representations to indicate normalization to clinical concepts. Concepts in the Unified Medical Language System (UMLS) Metathesaurus have a concept unique identifier (CUI) and a type unique identifier (TUI, i.e., semantic type) which are curated, normalized codes. For example, “pain” would have a UMLSConcept:cui = “C0030193” and UMLSConcept:tui = “T184”. Instead of recreating these ontological structures within the common type system, concepts are created by reference to any existing resource, as needed by an application.

Ontology concepts may exist, irrespective of whether they are instantiated in a particular clinical document. To capture and summarize what is actually indicated by a clinical text, we introduce refsem.Element, our foundational structure for deep semantics in the context of clinical care. An Element contains some of the same semantic information as IdentifiedAnnotation types, namely Element:polarity, Element:conditional, Element:uncertainty, Element:generic, and Element:subject. However, it assumes a single, disambiguated word sense to be set as an Element:ontologyConcept. Because multiple IdentifiedAnnotations in the text can refer to the same thing in the real world, there is an Element:mention array that allows quick access to mapped text. This illustrates the difference between the textsem and refsem namespaces; for example, a refsem.Event would aggregate all the features of co-referring textsem.EventMention objects. Note that some of the attributes may remain unused for subtypes of Element, such as the Element:polarity feature for Time and Date.

Similar to IdentifiedAnnotations, Elements have the non-temporal subtype refsem.Entity and the temporal subtype refsem.Event. In the constrained clinical context, most concepts are discussed as instances with some temporal component, e.g., Medications, Labs, and Diseases, while AnatomicalSites are Entities.

Other subtypes include Refsem.Time, which refers to a real-world time instance and stores a timestamp in Time:normalizedForm. Similarly, refsem.Date represents dates and puts them in a structured normalized form with Date:day, Date:month, and Date:year.

### Core clinical elements

Although the Element type is general enough to handle real world entities of many kinds, we take special effort to develop structure for things in the clinical domain. SHARPn has identified 6 general NLP-relevant CEMs that are here converted into types: refsem.AnatomicalSite, refsem.DiseaseDisorder, Refsem.Lab, Refsem.Medication, Refsem.Procedure, and Refsem.SignSymptom. These 6 groups are intended to house concepts from UMLS semantic groups, [16] with a few exceptions; Signs & Symptoms is a subgroup within Diseases & Disorders, Labs is a subgroup within Procedures, and Medications are the subset of Chemicals & Drugs that are present in RxNORM.

In the type system, these clinical elements primarily differ from each other in their attributes; for example, a “strength” feature as used in Medications would be irrelevant for Procedures.

### Core clinical attributes

The refsem.Attribute type forms the basis for post-coordination in our clinical semantics. In the current model, these types simply contain strings or numbers with units. Instead of strong typing (e.g., lab values must be “physical quantities” in some standards), default value sets are for the most part included in a file of constants that augment the type system. Again, not all Attributes apply to all Element types; also, some of the important features in the core clinical element types are not Attributes (e.g., SignSymptom:startTime). This means that the link between Attributes and their respective allowable values is not enforced by the type system itself; experience has shown us that hard-coded value sets tend to hamper development of downstream application-specific tools.

A few Attributes are common to multiple core CEMs. Refsem.BodyLaterality (medial vs. lateral, distal vs. proximal, etc.) and refsem.BodySide (left, right, bilateral) have to do with relative physical location. The progress or decline of a condition is marked through refsem.Course (changed, increased, decreased, improved, worsened, resolved, unmarked), and explicit indications of the seriousness of a condition are accounted as refsem.Severity (severe, moderate, slight, unmarked).

Procedures, Labs, and Medications each have more specific attributes, and these are further documented in the type system implementation.

### Relation types

**Figure 5:**
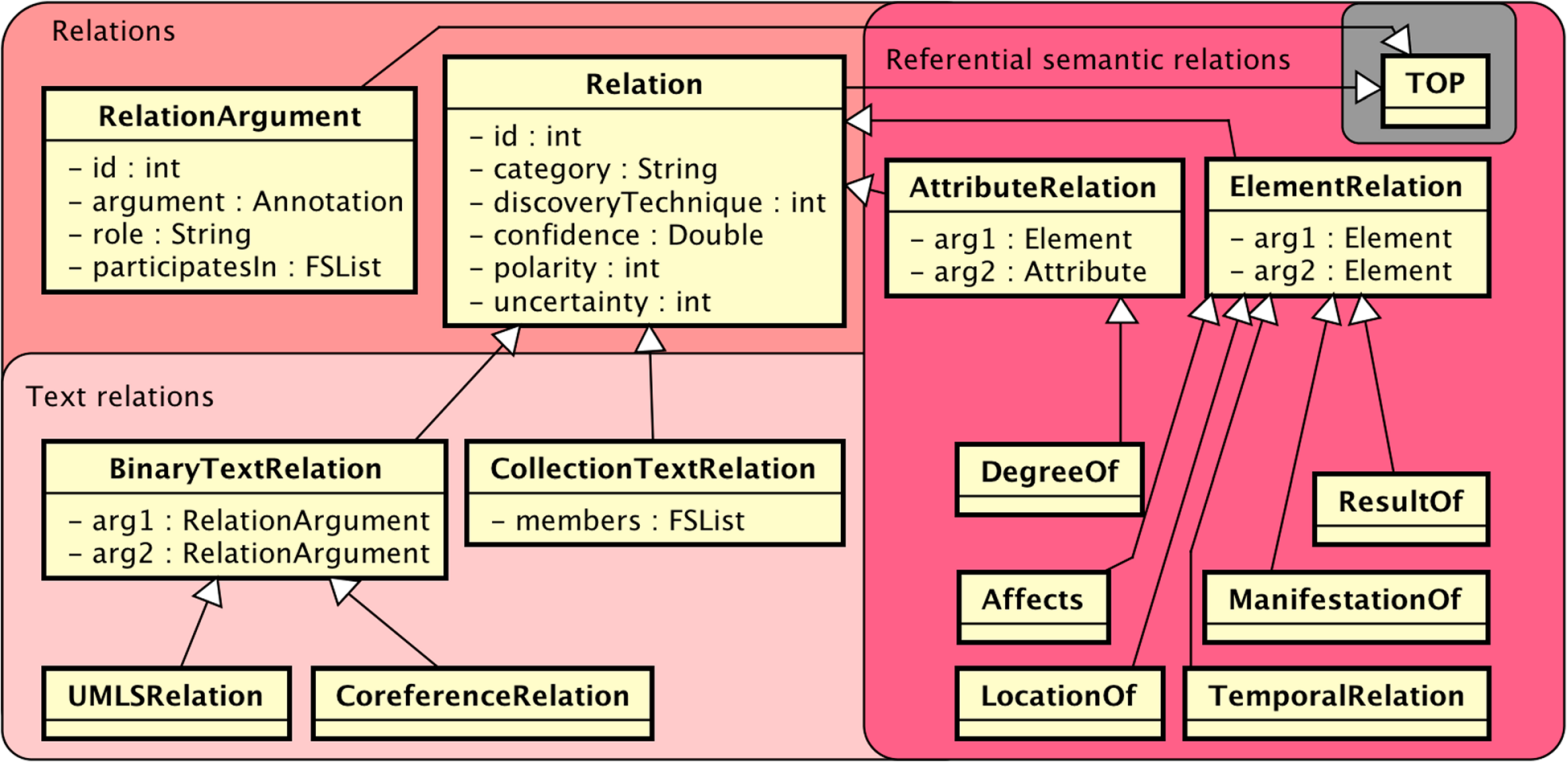
The relation namespace, with both text relations (spanned) and referential semantic (unspanned) relations.

Relation types in the common type system connect data structures at both the level of text semantics (connecting to the textsem namespace) and referential semantics (the refsem namespace). Shown in Figure 5, both of these are based on the relation.Relation type. Relations themselves are not tied to specific spans of text. The Relation type ensures that subtypes all have a “category” attribute (the label or semantic type of the relation), and the ability to represent negative (“polarity”) or uncertain (“uncertainty”) relationships. For example, “cancer has not spread to the lymph nodes” would yield a negative “locationOf” relationship.

Text relation subtypes (relation.BinaryTextRelation and relation.CollectionRelation) use the relation.RelationArgument type, which wraps an Annotation and therefore implies a text span. Subtypes of BinaryTextRelation, such as Relation.UMLSRelation, tie together two text spans and are further distinguished (e.g., when a medication “treats” a disease) via the inherited Relation:category.

Coreference (encapsulated in relation.CoreferenceRelation) is another text relation, and is particularly important because it bridges between the text semantics and referential semantics worlds by indicating that two text spans (mentions) actually refer to the same underlying real-world instance.

Relation.ElementRelation and relation.AttributeRelation extend the Relation type rather than the BinaryTextRelation type; thus, they operate without a spanned location. The types of relationships they are designed to express are those between real-world objects. For example, a coreference relation would be inappropriate for this type of relation, because it links multiple textual mentions as referring to the same entity. This type of relation might instead be a higher-level relationship between two such coreference-resolved entities.

ElementRelations are used for things like relation.TemporalRelation that link two Time, Date, or Event annotations. Other subtypes of ElementRelation include specific UMLS relations between referential semantic objects (rather than text spans): Relation.DegreeOf, Relation.Affects, Relation.LocationOf, Relation.ResultOf, and Relation.ManifestationOf.

### Statistics

As shown in Table 1, the defined common type system contains a total of 100 types and 207 attributes. This is expanded from 60 types in cTAKES v1.1 and 97 types in our preliminary common type system. 38 of the types are modified, 22 are deleted (from Use Cases), and 58 are newly defined as detailed in previous sections. The average number of attributes per type has also increased from 1.63 in cTAKES v1.1, to 2.07 attributes per type. The detailed semantic CEMs are a significant factor in this.

**Table 1:** Distribution of types in the common type system

**Type subdivisions**	**# of types**	**# of features**
**Structured**	4	24
**Syntax**	26	33
**RefSem**	31	96
**TextSem**	17	33
**TextSpan**	5	5
**Util**	3	3
**Relation**	14	13
**Total**	100	207

## Discussion

### Semantic pipeline example

The foregoing type system allows for NLP techniques to reach a depth of clinical semantic normalization that was not previously possible, and which we believe will be the primary gathering point for interoperable systems. We illustrate this semantic depth here by an example, which considers the clinical text: “Chief complaint: severe cough and fever. Cough started 2 days ago, no expectoration.”

**Figure 6:**
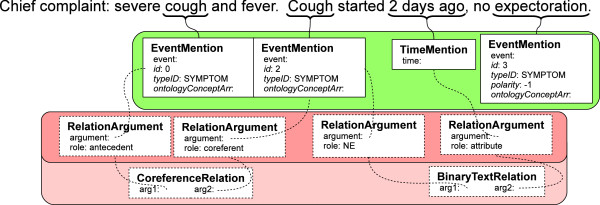
**Example results of NER and relation detection.** A shallow semantic representation with named entities and textual relationships. Boxes show instances of types from the common type system associated with the example sentence. For clarity, only relevant features with example-specific values are shown. Small black boxes refer to instances of non-primitive data types; the actual instances for EventMention:ontologyConceptArr.

Figure 6 shows semantic annotation that has typically been discovered in clinical NLP algorithms. In the figure, we have grouped instantiated types by their namespaces (text semantic types – green, relation types – red). Italicized features are inherited features, but many are omitted for clarity. Small black boxes indicate references to other, non-primitive data types. So, for example, CoreferenceRelation:arg1 has the leftmost RelationArgument as its value.

Text semantic types that would be found by some NER procedure would include EventMentions for both “cough” and “Cough.” Each of these would have a begin point and an end point in the text string, an identifier, a type, possible ontology normalizations via IdentifiedAnnotation:ontologyConceptArr, and possibly other features. A TimeMention would similarly be associated with “2 days ago,” and RelationArguments and a BinaryTextRelation would be used to identify that “2 days ago” is an attribute of “Cough.”

This level of semantic information is where many NLP systems stop. Most importantly, there is one real-world “cough” but two mentions. As a corollary, while the first mention is associated with “severe,” it does not immediately know about the temporal information of “cough.” Thus, without deeper semantics, this is insufficient to interoperate with structured data (e.g., for the SHARPn high throughput phenotyping effort).

**Figure 7:**
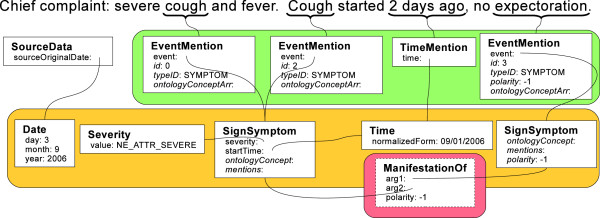
**Example results of deep semantic processing.** A deep semantic representation with coreferring mentions resolved, attributes combined, and a relationship inferred. The relevant SignSymptom:ontologyConcept instances (disambiguated concept identifiers) have been omitted. In this example, we have omitted the line from SignSymptom:mention to EventMention instances since they are implied by the links from EventMention:event to SignSymptom.

We show in Figure 7 the referential semantics that are not easily resolved without our type system. Here, the “cough” and “Cough” EventMentions have been resolved to a single “cough” SignSymptom type, ostensibly with severity specified, start time filled in, a disambiguated IdentifiedAnnotation:ontologyConcept, and backpointers to the mentions (links not shown for clarity). The Time type has been normalized to “09/01/2006” against the date of the document. Also, a referential semantic relationship shows that the “cough” is not manifesting itself with “expectoration.”

While this information is available in Figure 6, multiple links and resolution steps must take place before there is truly a clinical semantics that will interact correctly with structured data for high-throughput phenotying. In this common type system, we have provided a framework for including these crucial steps.

Practical applications may extend the type system beyond Figure 7. New local types may be created; for instance, creating a Cough type as a child of SignSymptom would allow an end application to consider all kinds of coughs (barking cough, whooping cough, etc.) jointly, but differentiated from other SignSymptoms. Additionally, use cases may be interested in introducing deep semantic templates parallel to CEMs that are not inherently clinical, such as GeographicLocation or Person.

### Related work

As mentioned, comprehensive NLP systems typically define the data structures they understand and populate. Thus, there are many examples of existing type systems, but the SHARPn NLP type system is unique in its covering of both structured semantics and lower-level features. HITEx [17] is GATE-based, and the majority of the types used there can be expressed within our type system (using syntax, text span, and text semantic types). The VA’s more recent UIMA-based v3NLP system builds off of this and additionally includes many SHARPn types. U-Compare is based on Colorado BioNLP types, which abstract the semantics away, whereas SHARPn types explicitly capture semantics of the mentions in CEM templates, as described below. Another type system is available through the JULIE Lab UIMA Component Respository (JCoRE) consists of NLP components developed by the JULIE lab and those available in the public domain. Our SHARPn type system aligns with JCoRE with respect to syntax, mention, and text span types. MedKAT [18] contains specialized structures to capture named entities from pathology notes; these structures are left to be locally defined in the SHARPn type system.

It should be noted that our proposed CEM-based semantic structure differs from the ontology representations or NER output that are present in many of these existing type systems. Previous type systems represented semantics as simply a mapped ontology code, or the named entity text along with semantic type. Ontology-mapped text is supported in our type system, but it shallowly defines the meaning and lacks the ability to capture attributes of a concept or to normalize multiple instances of the same concept. Instead, the SHARPn type system includes a highly-refined deep semantic structure, allowing for post-coordination and nuance. Some systems like MedLEE (Medical Language Extraction and Encoding System), ONYX, and MetaMap have included well-developed types for domain semantics [19-22]. These systems are well tuned for their own purposes, but often leave out other structures (e.g., semantic role labels) tied to more advanced NLP tasks.

## Conclusion

We have presented a comprehensive type system for Clinical NLP – a specification of data types with an end goal of deep computational semantic processing. The semantic representation presented here is a conglomeration of semantic models, especially Clinical Element Models. Additionally, elements of previous type systems and language processing standards for preprocessing, morphology, and syntax are included in the type system.

We have illustrated by example the means by which deep semantics can be obtained in this type system. Multiple possible methodological architectures are compatible with this type system. It is hoped that this emerging, practical type system will be used by the community at large as it provides a nurturing context for a diversity of activities in Clinical NLP.

## Endnotes

^a^See http://www.ldc.upenn.edu/Catalog/docs/LDC95T7/cl93.html.

## Abbreviations

SHARP: SHARPn, SHARP 4: Strategic healthcare IT advanced research projects, area 4; EMR: Electronic medical record; NLP: Natural language processing; CEM: Clinical element model; UIMA: Unstructured information management architecture; cTAKES: Clinical Text Analysis and Knowledge Extraction System; CAS: Common analysis structure (in UIMA).

## Competing interests

The authors declare that they have no competing interests.

## Authors’ contributions

SW, VK, DD, WW, GK, and HL specified the type system graphically and SW, JM, PC, LB, and HL implemented the type system in UIMA. CC conceived of the concept, SW drafted the manuscript, and all authors reviewed and edited the manuscript.
